# Dental Colleges

**Published:** 1869-03

**Authors:** E. D. Hamner


*Dental Colleges.*—Galveston Texas, January, 16th. Editor of The Weekly Star:

MR. EDITOR—Will you through the medium of your paper, allow me to correct an assertion made by the New Orleans correspondent of the "Galveston Dispatch"—that the New Orleans Dental College was the only institution of the kind in the South. He cannot be conversant with the progress of Dental Science or with the location of its institutions of learning. And in honor of my old Alma Mater, as a debt of gratitude to those old patriots who compose its faculty, I make this correction The Baltimore College of Dental Surgery was the first institution of the kind established on this continent and the first in the known world, so far as we have any knowledge. And to the South, and to the natives of the South, belongs the honor of first establishing a school where this noble science, so necessary and essential to the happiness of the human family, might be thoroughly taught and understood.

This institution was chartered by the Legislature of Maryland nearly thirty years ago. The present is its twenty-ninth annual session. And for the edification of said correspondent I will further state, that all the members of the faculty of this institution are natives of the South, and that no less than four of the present faculty were down South assisting the so-called rebels, during our unhappy struggle for independence.

Very respectfully, E. D. HAMNER, D.D.S.

We thank Dr. Hamner for so nobly sustaining his *Alma Mater*, and trust that the *Baltimore College of Dental Surgery* may ever merit the support of her alumni. It is very encouraging to those who devote health, time and labor to the cause of dental education, to know that our former pupils are ever ready to guard the honor and standing of the institution whose diploma they bear.

